# Effect of Simulated Low-Load Functional Loading on Voids Volume and Distribution of Different Retrograde Filling Materials: A Micro-Computed Tomography Analysis

**DOI:** 10.3390/jfb17020082

**Published:** 2026-02-08

**Authors:** Hanan Alharbi, Ezdyan Alsemanni, Areej Almutairi, Ali Alrahlah, Wafaa Khalil

**Affiliations:** 1 Department of Conservative Dental Sciences, College of Dentistry, Qassim University, Buraydah 52571, Saudi Arabia; 2 Endodontic Resident, Department of Conservative Dental Sciences, College of Dentistry, Qassim University, Buraydah 52571, Saudi Arabia; 3 Bachelor of Dental Surgery, College of Dentistry, Qassim University, Buraydah 52571, Saudi Arabia; 4 Department of Restorative Dental Sciences, Engineer Abdullah Bugshan Research Chair for Dental and Oral Rehabilitation, College of Dentistry, King Saud University, Riyadh 11545, Saudi Arabia

**Keywords:** endodontics, bioceramics, ERRM, MTA, microsurgery, filling ability, voids, in vitro study

## Abstract

Introduction: The outcome of endodontic microsurgery depends on the integrity of the apical seal and the adaptation of root-end filling materials under functional stresses. The study aims to compare the void volumes and distribution of ProRoot MTA, ERRM, and ERRM combined with Bioceramic sealer under simulated functional loading using micro-computed tomography (micro-CT). Methods: Forty-four single-rooted mandibular premolars were prepared with 3 mm apical cavities and divided into four groups (*n* = 11 each): Cavit (Control), ProRoot MTA, ERRM Putty, and ERRM + BC Sealer. Samples were scanned by micro-CT to quantify internal, marginal, and total voids. Each specimen was then subjected to cyclic vertical loading of 20 N for 1,000,000 cycles in a chewing simulator, followed by post-scanning. Pre- and post-loading void volumes and distribution were analyzed and compared statistically (α = 0.05). Results: Functional loading significantly increased void volumes in all groups (*p* < 0.05). Control and MTA showed the highest total and marginal voids (*p* < 0.05), while ERRM and ERRM + BC maintained significantly lower overall and marginal voids. No difference was detected between ERRM and ERRM + BC (*p* > 0.05). ERRM and ERRM + BC Sealer showed relatively lower marginal-to-internal voids ratios compared to MTA. Material dislodgement occurred only in Cavit and MTA. Conclusions: ERRM and ERRM + BC sealer groups exhibited favorable marginal adaptation and significantly lower overall void volumes after low-load functional loading compared to MTA and the control. The findings indicate preserved sealing performance and suggest resistance to void formation under simulated occlusal stresses.

## 1. Introduction

Successful microsurgical root canal treatment requires creating a stable, three-dimensional apical seal within the canal [1]. Retrograde filling materials used in such cases should exhibit biocompatibility, radiopacity, easy handling, resistance to breakdown, and promote periapical tissue regeneration [2]. Additionally, root-end filling materials must possess sufficient physical properties, such as dimensional stability, to maintain the integrity of the apical seal over time [3]. Hence, inadequate sealing of the apical root canal, including the presence of voids or gaps, can lead to microleakage, adversely affecting treatment outcomes and potentially resulting in failure [4,5,6]. Inadequate sealing of the apical root canal can lead to persistent periapical pathologies, as unfilled spaces can serve as reservoirs for microbial contamination and their toxic byproducts [7].

ProRoot MTA (Dentsply Sirona Endodontics, York, PA, USA) and EndoSequence Root Repair Material (ERRM, Brasseler, Savanna, GA, USA) are calcium silicate materials and among the most commonly used root-end filling materials, with clinical studies reporting high success rates [8,9]. Recent studies support the preferential use of these calcium silicate-based materials over amalgam and Super-EBA for root-end fillings due to their superior clinical outcomes, biocompatibility, and physical properties [10,11]. Both MTA and ERRM demonstrate effective resistance to bacterial and endotoxin penetration and maintain stable sealing over time [12,13,14,15]. Reports on leakage performance show variable findings, with studies indicating comparable outcomes as well as differences between the two materials after setting [13,16].

Recent studies have explored the combined use of calcium silicate-based cement with a bioceramic sealer, showing improved void reduction in the combined group versus cement alone [17]. This approach, known as the lid technique, involves the use of an injectable bioceramic sealer (BC, Brasseler, Savanna, GA, USA) followed by application of putty (ERRM; Brassler USA, Savannah, GA, USA). Clinical outcome studies have shown comparable success rates using this method without significant differences when compared to MTA or ERRM putty alone in retrograde restorations [18,19,20]. Another recent micro-CT research has demonstrated that hydraulic calcium silicate-based materials may undergo measurable volumetric changes depending on their set condition and exposure to fluids [21].

The effects of occlusal forces on retrograde fillings are also significant. Masticatory forces apply functional cyclic loading on teeth, which can impact the stability of filling materials [22,23]. Physiological masticatory forces in posterior teeth are substantially higher than the loads commonly applied in laboratory loading simulations. In vivo and clinical measurements have reported mean bite forces in the premolar and molar regions ranging between approximately 70 and 150 N, with some individuals able to reach up to 200–400 N under maximum voluntary clenching [24,25]. These forces reflect the functional biomechanical environment experienced by posterior teeth during mastication and parafunctional activities. However, in vitro chewing simulators frequently employ reduced loading magnitudes (e.g., 20–100 N) to prevent catastrophic specimen failure and allow controlled fatigue testing [26]. While such loads facilitate experimental standardization, they may underestimate the mechanical demands encountered clinically, potentially limiting the translational validity of laboratory findings. Recent biomechanical and finite element analyses emphasize the importance of incorporating graduated load levels (50–400 N) to better approximate physiological and parafunctional occlusal forces and to characterize material behavior across elastic and fatigue thresholds [27,28].

Studies have shown that masticatory loads are significantly associated with increased apical leakage [29]. Experimental models further indicate that relatively low occlusal forces, within the range of 20–117 N, are sufficient to induce measurable mechanical consequences at the apical region during mastication [30]. Other research has explored how different pressure levels starting at 20 N impact the periapical tissues [31]. The magnitude of masticatory forces may vary according to patient-related factors such as age, sex, diet, and muscular strength [32]. However, finite element studies suggest that under normal masticatory loading, stress concentration at the root apex is unlikely to be affected in either intact or instrumented teeth [33]. In contrast, chewing simulation studies comparing retrograde filling materials have demonstrated that while materials such as MTA and EBA generally maintained stable apical seals, minor changes in marginal adaptation may occur following cycling loading [34].

Despite advances in retrograde filling materials, the effect of loading on teeth with such fillings remains overlooked. Particularly, the impact of combining a bioceramic sealer with ERRM has not been thoroughly investigated. Therefore, this study aims to compare the void volumes and distribution of ProRoot MTA, ERRM, and ERRM combined with Bioceramic sealer under simulated low-load functional loading conditions using micro-CT, to assess their sealing ability and material adaptation. The null hypothesis is that there are no significant differences in void volume or distribution among the tested retrograde filling materials under simulated functional loading.

## 2. Materials and Methods

The study was approved by the Committee of Research Ethics in Qassim University no. (23-20-08). The methodology was designed according to PRILE2021 guidelines [35]. Forty-four single-rooted human mandibular premolars were collected after being extracted for orthodontics reasons. All patients have signed the informed consent form, agreeing to the use of extracted teeth for scientific research. Two periapical radiographs were taken for examination. Teeth with single root canal morphology and, with absence of periapical lesions were included. Teeth with more than one canal, previous root canal treatment, calcifications, immature apices, internal resorption, fractured or decayed were all excluded. The sample size estimation (*n* = 11 per group) was determined based on previous in vitro micro-CT studies with comparable designs and confirmed by an a priori power analysis

### 2.1. Sample Preparation

Access opening was performed for all teeth and working length was determined 0.5 mm short of the apical foramen. Canals were instrumented using ProTaper Next system (Dentsply Maillefer, Ballaigues, Switzerland) up to size 40 (X4). Canals were irrigated using 2.5% sodium hypochlorite solution then finally flushed with EDTA. Thereafter, the canals were obturated using gutta percha cones with AH plus sealer. Then all teeth were sealed coronally with a permanent composite restoration. Final periapical radiographs were taken to confirm filling.

### 2.2. Retrograde Preparation and Filling

Under the dental operating microscope (Zeiss, Oberkochen, Germany), root-end resection of 3 mm was performed, ensuring 90° to the longitudinal axis of the tooth, and using Lindemann Bur (Brasseler USA, Chicago, IL, USA). Retrograde preparation of 3 mm was achieved using an ultrasonic tip (ED10D, Zumax, Suzhou, China). To standardize the root-end preparations, a microplugger with a 1.0 mm tip diameter was inserted 3 mm into each canal to confirm a consistent cavity dimension. After that, teeth were randomly allocated into four groups according to the retrograde filling material (*n* = 11 each). Group (1) received temporary filling Cavit (Cavit; 3M ESPE, Seefeld, Germany) and served as Control. Group (2) received ProRoot MTA. Group (3) received ERRM, while Group (4) received ERRM + BC sealer. Cavit was included as a negative functional control to validate the sensitivity of micro-CT in detecting void formation under loading conditions.

Each tooth was labeled with a specific sample number. All filling materials were handled according to the manufacturer’s instructions. For the control group, the cavities were filled with temporary filling material Cavit and condensed with a microplugger. In Group 2 and 3 samples, ProRoot MTA and ERRM Putty were placed into the retrograde cavities using a microplugger and compacted incrementally. In Group (4), a combination of ERRM Putty and BC Sealer was used. The BC sealer was first injected into the retrograde cavity to coat walls, followed by application of ERRM Putty.

After completion of the retrograde fillings, periapical radiographs were taken to confirm the quality and adaptation of the materials. All specimens were then stored in 100% relative humidity for 7 days before being sent to pre–micro-CT scanning.

### 2.3. Micro-CT Scan

Samples were then loaded and mounted into the specimen chamber guided by a pre-established position in the micro-stage in order to produce a reproductible representation of pre- and post data sets.

Micro-CT scanning was acquired for the numbered specimens (SkyScan1172, Bruker SkyScan, Kontich, Belgium). Scanner settings set to capture an oversized scan were configured to use a 1048 × 2000 image matrix, 96 kV voltage, 102 µA anode current, 1180 ms exposure time, 6.91 µm image pixel size, Al + Cu filter, 0.5 rotation step for 180° angle capturing 410 projections, frame averaging of 4 for improved signal to noise ratio and random movement of 8 minimize ring artifacts. A Flat-field correction was performed before the scanning procedure in order to correct variations in the camera pixel sensitivity.

After the scanning, a reconstruction of the projected images was performed using ©N-Recon, program version 1.6.9.4 (Bruker Skyscan, Kontich, Belgium) to produce a reconstructed cross-section images. Numerical parameters needed to establish the best image results were checked and adjusted. A ring artifact reduction of 5 for non-uniformity of the background image taken by the X-ray camera; 25% beam hardening compensation to prevent the specimen from appearing artificially denser at or near its surface, and less dense at its central parts; and a smoothing of 2 using Gaussian kernel were applied.

Reconstructed images were loaded to the ©Dataviewer program version 1.5.6.2 (Bruker Skyscan, Kontich, Belgium) Software to determine image quality, reorient, resize and visually inspect sample 3D image data sets. A trans-axial data set was saved to be used for analysis.

### 2.4. Functional Loading

**Figure 1:**
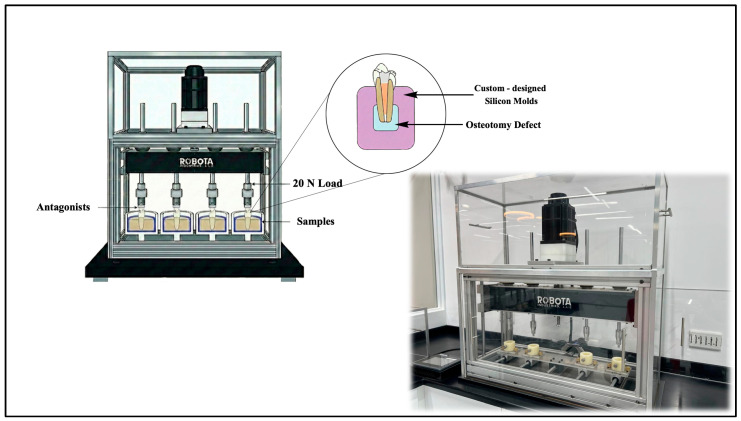
Photograph and schematic illustration of the chewing simulator device and experimental setup.

Teeth were embedded in custom-designed silicone molds mimicking the periapical tissues, periodontal ligament and surrounding osteotomy defect with a 5 mm diameter empty space around the resected apex (Figure 1). Vertical cyclic loading was applied on crowns using a chewing simulator (Robota, third Industrial Area, Unit 13, Block 16, New Borg El Arab, Alexandria) equipped with opposing acrylic maxillary premolars as antagonists. The simulation was performed at 20 N load, with a 4 mm displacement, 120 cycles/min, for a total of 1,000,000 cycles. This was calculated to equal approximately 4 years of low-load functional mastication under typical oral daily conditions. Based on commonly used assumptions in chewing simulation studies, approximately 240,000–250,000 cycles have been reported to correspond to one year of clinical function [36,37]. Following simulation, specimens were subjected to post micro-CT scanning to quantify void volumes and distribution. Post-Miro-CT scans were taken for all groups following the same specifications as the Pre-Micro-CT scan.

### 2.5. Analysis and Imaging

Pre- and post trans-axial data sets were then loaded in the ©CTAn version 1.20.8.0 software for imaging and analyzing selectively, binarizing and quantifying pre- and post scan samples’ region of interest. Segmentation was performed using a global thresholding approach based on gray-scale histogram inspection and visual calibration to differentiate dentin, filling material, and void spaces. The same threshold settings were applied consistently across all specimens within each scanning session. Internal voids were defined as voids completely enclosed within the filling material, whereas marginal gaps were defined as voids in direct contact with the material–dentin interface. The volumes of voids (overall, internal and gap) were calculated based on previous studies [17], using the following formulae:

%V_out_ = V_out_/(V_out_ + V_in_ + V_m_) × 100

%V_in_ = V_in_/(V_out_ + V_in_ + V_m_) × 100

%V_total_ = V_out_ + V_in_/(V_out_ + V_in_ + V_m_) × 100

Gray-scale thresholds were defined to segment different volumetric components: values between 56 and 255 were assigned to the filling material volume (Vm), values from 0 to 10 corresponded to the interfacial gap between the filling and the root canal wall (Vout), and values between 0 and 56 represented internal voids within the filling (Vin). Sequential micro-CT slices for each specimen were subsequently reviewed, and gray-scale thresholds were adjusted when necessary to correct potential segmentation inaccuracies.

### 2.6. Statistical Analysis

Data were analyzed using SPSS version 20 (SPSS Inc., Chicago, IL, USA). Normality was assessed prior to analysis. As the data were not normally distributed, non-parametric tests were applied. Comparisons among the four groups were performed using the Kruskal–Wallis test. When significant differences were detected, pairwise comparisons were conducted using the Mann–Whitney U test with Bonferroni correction for multiple comparisons. Pre- and post-loading comparisons within each group were performed using the Wilcoxon signed-rank test. Effect size for overall group differences was calculated using eta-squared (η^2^). Statistical significance was set at *p* < 0.05.

## 3. Results

**Figure 2:**
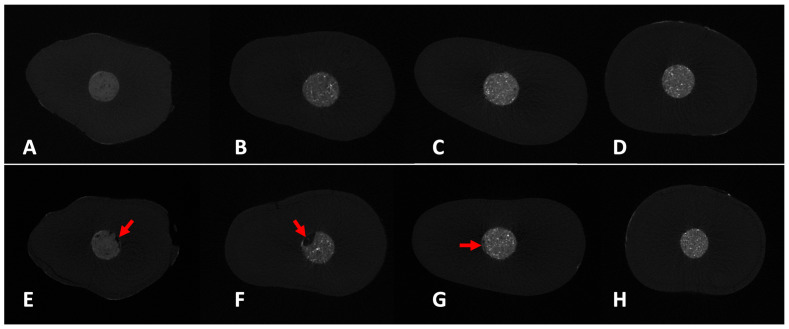
Representative trans-axial micro-CT images showing retrograde fillingmaterials before (upper row: **A**–**D**) and after (lower row: **E**–**H**) functional loading. (**A**,**E**) Control group; (**B**,**F**) ProRoot MTA group; (**C**,**G**) ERRM group; (**D**,**H**) ERRM + BC Sealer group. Red arrows indicate voids identified at the margin (material-dentin interface) and within the retrograde filling.

The total volume of voids significantly increased after funcational loading across all groups (*p* < 0.05). In both stages, the control group exhibited significantly higher overall void volumes compared to all other groups (*p* < 0.05). Effect size analysis using eta-squared (η^2^) revealed large effects for overall void volume and marginal gap in the post- loading stage (η^2^ = 0.48–0.52), and a large effect for internal voids in the pre-loading stage (η^2^ = 0.59). Moderate effect sizes were observed for overall voids and marginal gaps in the pre-loading stage. MTA had significantly higher overall voids post-loading compered to both ERRM and ERRM + BC sealer groups (*p* < 0.05). There was no statistically significant difference between ERRM and ERRM + BC Sealer groups, in either the pre- or post-loading stages (*p* > 0.05). Overall, the ERRM and ERRM + BC sealer groups consistently demonstrated lower volume of voids, both internally and at the margins, before and after loading simulation (Table 1 and Table 2) (Figure 2).

After functional loading, all groups exhibited higher void accumulation at the margins compared to internal regions. Control and MTA groups showed significantly the most pronounced marginal dominance (*p* < 0.05), while ERRM and ERRM + BC Sealer showed relatively lower marginal-to-internal voids ratios (Table 3).

**Figure 3:**
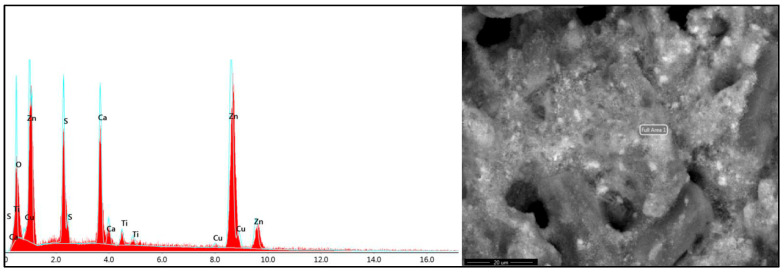
Energy-dispersive X-ray spectroscopy (EDX) and SEM micrograph of the dislodged Cavit fragment. The spectrum exhibits dominant zinc (Zn) peaks with sulfur (S), oxygen (O), and minor calcium (Ca), consistent with zinc-oxide-based temporary filling material. The SEM micrograph reveals a porous, irregular surface structure indicative of Cavit disintegration under functional loading.

**Figure 4:**
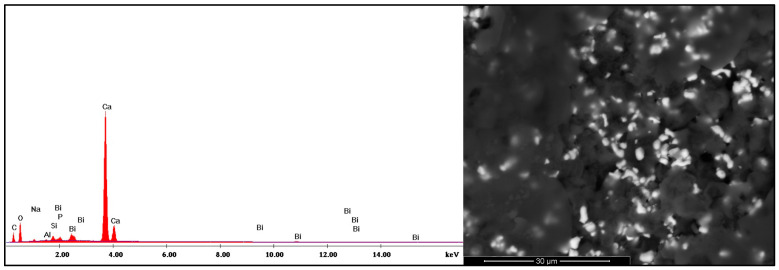
EDX spectrum and SEM micrograph of the dislodged ProRoot MTA fragment. The elemental analysis shows a Ca-Si-O-rich matrix.

During disassembly of the samples after chewing simulator, remnants of dislodged material were detected within the simulated periapical lesion/osteotomy area in the MTA and Cavit groups. These remnants were examined using scanning electron microscopy and elemental analysis to verify their composition, which corresponded to the structural characteristics of the original retrograde filling materials (Figure 3 and Figure 4).

## 4. Discussion

The clinical success of endodontic microsurgery is not only determined by material selection or sealing ability in dry, controlled conditions. Rather, it can be identified by how well the apical interface withstands the dynamic interaction of occlusal forces, biological fluids, and tissue responses. This perspective challenges the conventional static evaluation of retrograde materials, and emphasizes the need to assess performance under functional load. In the current study, we investigated the sealing ability and volumetric voids distribution of different retrograde fillings subjected to simulated low-load functional forces. The findings showed that ERRM and ERRM + BC sealer groups exhibited significantly lower void volumes after functional loading compared to MTA and control groups. This behavior may be attributed to the intrinsic properties of ERRM materials, including improved handling and washout resistance, which may promote more uniform adaptation and enhanced interfacial stability under cyclic mechanical stress compared with MTA. The observed large effect sizes indicate that the differences among materials were not only statistically significant but also of practical relevance. This highlights the preserved sealing performance of these materials and suggests resistance to void formation under simulated loading.

Functional chewing forces generate cyclic mechanical loading that can compromise the long-term stability of dental materials, particularly through microcrack propagation and interfacial degradation [38]. While these effects are well documented in coronal restorations, their impact on retrograde fillings are not well studied. Functional loading was performed using a vertical-loading device that applied repeated low-magnitude compressive forces through acrylic antagonists, aiming to reproduce cumulative masticatory stresses over time.

The current study employed a 20 N cyclic loading force, which represents only a fraction of physiological posterior bite forces reported in vivo, typically ranging approximately 70 to 400 N [24,25]. Although such reduced loads are commonly used in laboratory fatigue protocols to preserve specimen integrity and permit longitudinal volumetric assessment, this magnitude does not fully replicate functional occlusal stresses encountered in clinical mastication. Furthermore, the applied model does not replicate the full complexity of intraoral conditions, including oblique and lateral forces. Nevertheless, it provides a clinically relevant estimate of sub-occlusal impact on retrograde filling materials.

The applied load (20 N) was intentionally selected to represent low-load repetitive forces rather than peak masticatory loads. This approach has been previously adopted to evaluate early mechanical fatigue and interfacial stability without inducing catastrophic material failure [26,39,40]. Biomechanical studies have demonstrated that posterior occlusal loading exhibits a nonlinear dose–response behavior, in which incremental increases in load (e.g., 25, 100, 200, 300, and 400 N) result in progressive stress amplification and distinct mechanical failure patterns [28,41]. Furthermore, fatigue and chewing-simulation investigations suggest that higher load gradients improve the relevance of experimental models and allow identification of the linear elastic threshold beyond which irreversible structural damage and interfacial degradation occur [39]. Accordingly, a substantial difference remains between the maximum physiological masticatory forces that can be exerted in the posterior region and the lower loads typically applied in laboratory functional loading simulations, which should be considered when extrapolating in vitro findings to clinical performance.

The so-called “lid technique”, which combines a flowable bioceramic sealer with a putty-type calcium silicate cement, has been proposed to enhance adaptation of retrograde fillings. However, interpretation of its effect on void formation depends strongly on the outcome parameter assessed. Tran et al. reported improved marginal adaptation for a sealer–putty combination at a specific coronal level using SEM-based gap distance measurements, while no significant differences were observed at the anatomic apex. Importantly, that study did not evaluate internal or total void volume using micro-computed tomography [13]. In contrast, Jung et al. directly quantified internal voids, interfacial gaps, and total void volume using micro-CT and reported numerically lower void values for calcium silicate putty used alone compared with the sealer–putty combination, although without statistical significance [17]. The present study is methodologically more comparable to the latter, as void volume was directly assessed using micro-CT. Our findings demonstrated a similar trend during the pre-loading stage, while after simulated loading, the ERRM + BC Sealer group exhibited a numerical reduction in void volume without statistical significance. These observations suggest that the influence of the lid technique on void formation may be stage-dependent and influenced by material properties and evaluation timing rather than representing a uniform reduction in void volume. This methodological distinction is further supported by recent micro-CT investigations of calcium silicate–based materials, in which internal voids and interfacial gaps were treated as independent outcome parameters rather than interchangeable indicators of sealing quality [42].

Post-simulation micro-CT scans revealed a consistent increase in void volume across all tested groups, suggesting that vertical mechanical loading can compromise the marginal adaptation of apical materials [34]. This is particularly evident at the dentin–material interface, where fatigue-related microgaps may propagate. ERRM and ERRM + BC sealer demonstrated significantly lower marginal gap values than MTA following functional loading simulation, with no statistically significant difference between the two bioceramic groups, consistent with previous static reports [17,43]. Recent literature emphasizes that reproducibility in dental micro-CT studies relies primarily on transparent reporting of voxel size, energy source, fixation method, and thresholding approach, rather than adherence to a single formal standard [21,42,44,45,46,47]. Although no specific ASTM standard exists for micro-CT evaluation of voids in endodontic filling materials, the scanning, reconstruction, segmentation, and reporting procedures in the present study followed widely accepted and standardized micro-CT practices for dental materials research, as described in recent reports [13,17,21].

The immediate microenvironment following surgical root-end treatment is characterized by the presence of blood, extracellular fluids, and inflammatory mediators, which may significantly alter the physicochemical behavior of retrograde filling materials. Unlike isolated standardized in vitro conditions, these factors interact with hydration kinetics, setting reactions, and interfacial bonding of calcium silicate-based materials [48]. Voids indicate micro-disintegration or detachment of particles from the material surface. This was observed upon disassembly of the simulated periapical tissues in this study, where small fragments appeared dislodged, especially in control and MTA samples. This is in line with a previous report by Khalil et al. (2015), who demonstrated reactions in adjacent tissues induced by the detached particles from calcium silicate-based materials [49]. Further investigation into the local and possibly systemic behavior of detached microfragments under clinical conditions is required.

In the present study, all materials were allowed to fully set under humidified conditions before functional loading simulation. This simulated the clinical scenario where root-end fillings are exposed to a biologically moist environment prior to functional stress. Post-simulation micro-CT analysis revealed that while all groups with fully set materials exhibited some increase in void volume, the ERRM + sealer group demonstrated the lowest overall void volumes and less marginal gap, both before and after functional simulation. This is in alignment with Enkhbile et al., (2024), who reported that lid technique was faster and less prone to nanoleakage when compared to MTA [50]. Other clinical studies also have shown successful results of lid technique compared to other retrograde materials [18,19]. Furthermore, several studies have shown that microleakage predominantly occurs along the marginal interface between the retrofilling material and dentinal walls, which represents the weakest point of the root-end seal [50,51]. Overcoming the high susceptibility of these margins to stress concentrations and microleakage might be better addressed with the superior adaptability and lower void ratio observed in the sealer added group.

The findings of our study align with previous evidence suggesting that hydraulic cements vary in volumetric stability depending on their set condition and interaction with surrounding fluids [21]. Sinsareekul et al. [21] demonstrated that incompletely set MTA undergoes significantly greater volumetric reduction compared with fully set MTA, indicating its susceptibility to early dissolution when exposed to moisture. Our results expand on this by showing that, beyond volumetric change, functional loading further influences void behavior and material integrity, an aspect not evaluated in previous static models.

This in vitro study has some limitations. It has simulated vertical loading with only 20 N, representing a minimal approximation of sub-occlusal forces and does not reproduce the complex cyclic and multidirectional stresses, with higher magnitude of forces in vivo. Biological factors such as blood fluids and tissue interactions were not replicated in this model. Micro-CT allowed void quantification but not chemical degradation. Also, due to the voxel size (6.91 µm), possible nanoscale defects smaller than the resolution limit, were not assessed.

## 5. Conclusions

ERRM and ERRM+ BC sealer groups exhibited favorable marginal adaptation and significantly lower overall void volumes after the low-load functional loading simulation compared to MTA and the control. The findings indicate stable sealing performance and suggest resistance to void formation under simulated occlusal stresses.

## Figures and Tables

**Table 1:** The percentage volumes for overall voids (%V_total_), internal voids (%V_in_) and the gap between the retrograde filling and the canal wall (%V_out_), and in canals with retrograde fillings in all 4 groups (*n* = 11 each) in the pre-loading stage.

Variable	Median % (QR)				*p* Value
	Control	MTA	ERRM	ERRM + BC Sealer	
Overall Voids (%V_total_)	8.99 (2.76)	3.72 (3.87)	3.46 (1.63)	4.39 (2.54)	0.009 *
Internal voids (%V_in_)	1.75 (0.62)	0.27 (0.36)	0.69 (0.16)	0.78 (0.74)	0.000 *
Gap (%V_out_)	5.01 (2.40)	3.20 (3.58)	2.77 (2.04)	3.19 (1.81)	0.058

QR: Interquartile range (Q3-Q1). * Significant difference among all the tested groups (*p* < 0.05).

**Table 2:** The percentage volumes for overall voids (%V_total_), internal voids (%V_in_) and the gap between the retrograde filling and the canal wall (%V_out_), and in canals with retrograde fillings in all 4 groups (*n* = 11 each) in the post-loading stage.

Variable	Median % (QR)				*p* Value
	Control	MTA	ERRM	ERRM + BC Sealer	
Overall Voids (%V_total_)	11.99 (1.81)	10.61 (5.02)	4.63 (1.94)	4.58 (0.92)	0.000 *
Internal voids (%V_in_)	1.58 (0.05)	1.57 (1.16)	1.13 (0.62)	1.19 (0.18)	0.042 *
Gap (%V_out_)	10.85 (1.66)	9.18 (5.01)	3.40 (1.29)	3.18 (1.18)	0.000 *

QR: Interquartile range (Q3-Q1). * Significant difference among all the tested groups (*p* < 0.05).

**Table 3:** Relative distribution of voids post-loading (% of total void volume within each group).

Group	Internal Voids (%)	Marginal Gaps (%)
Control	12.7%	87.3%
MTA	14.6%	85.4%
ERRM	24.9%	75.1%
ERRM + BC Sealer	27.2%	72.8%

## Data Availability

The original contributions presented in the study are included in the article, further inquiries can be directed to the corresponding author.
